# An unusual clinical presentation of a plunging ranula—The plunging ranula with extension to the vallecula

**DOI:** 10.1002/ccr3.8964

**Published:** 2024-06-14

**Authors:** Lisa Schmitz, Franziska Büscheck, Christian Stefan Betz, Arne Böttcher

**Affiliations:** ^1^ Department of Otorhinolaryngology University Hospital Hamburg‐Eppendorf Hamburg Germany; ^2^ Department of Pathology University Hospital Hamburg‐Eppendorf Hamburg Germany

**Keywords:** marsupialization, oral mucocele, plunging ranula, vallecular cyst

## Abstract

**Key Clinical Message:**

A plunging ranula may present initially as an extensive vallecular cyst and correct diagnosis may be reached with the use of ultrasound, fluid aspiration for amylase detection, and MRI imaging.

**Abstract:**

The ranula is a pseudocyst of the sublingual salivary gland and can be divided into two known subtypes. The simple ranula and plunging ranula. While the simple type can be found in the floor of the mouth, the plunging ranula usually pervades the mylohoid muscle and presents as a cervical swelling. The presented case should outline the difficulties in diagnostic and treatment of an uncommon expression of a mucocele above the mylohoid muscle without presenting either a cervical or an intraoral swelling, only extending towards the vallecula. We present a previously unreported clinical manifestation of a ranula of an 18‐year old male, which extends posteriorly, remaining confined in the supramylohyoid muscle space. The cystic lesion protrudes in the oropharynx, and clinically appears as an extensive vallecular cyst. On magnetic resonance imaging the initial suspected diagnosis of a vallecular cyst was changed to the final diagnosis of a plunging ranula. The marsupialization of the cyst sac was performed. Outpatient follow‐up revealed a persisting ostium, indicating a continuous extravasation of the sublingual gland. The present case report describes an unusual clinical presentation of a plunging ranula, remaining above the mylohyoid muscle and protruding into the oropharynx, misdirecting to the first suspected diagnosis of a vallecular cyst. The case highlights the useful contribution of the MRI imaging for differential diagnoses and the need for criteria to indicate further investigations.

## 1 BACKGROUND

The ranula is an extravasation cyst of the sublingual gland that is usually caused by trauma with a consecutive tearing of the duct of Rivinus.^1^, ^2^ The emerged mucocele presents commonly a pseudocyst with a nonepithelial lining of the cavity.^3^, ^4^, ^5^

The clinical presentation can be divided into two types. The intraoral or simple ranula usually presents as a livid, cystic mass on the floor of the oral cavity, lateral to the lingual frenulum and confined to the supramylohyoid space.^6^ It leads to dysphagia or throat lump,^7^ usually appears unilateral, and children and young adults seem to be more affected.^8^

The plunging ranula is a mucocele^9^ with an hourglass‐shaped herniation through the mylohyoid muscle into the submandibular region, mainly due to a defect in the corresponding muscle, presenting as cervical swelling.^6^, ^10^, ^11^ Occasionally, it leaves the sublingual region via the posterior margin of the mylohyoid rather than through a mylohyoid hiatus.^12^ In addition to injury to the glandular excretory duct, a genetic predisposition has been suggested.^13^ The plunging ranula may or may not have an oral component.^3^

Since spontaneous remission is rarely observed, current literature agrees on the performance of a treatment. The preferred procedure for the simple and plunging ranula is the complete removal of the affected gland, either via a transoral approach alone or in cases of cervical manifestations of plunging ranulas in combination with a transcervical approach.^14^, ^15^, ^16^ For the simple intraoral ranula, marsupialization provides a less invasive technique and is therefore still frequently used.^2^, ^4^, ^7^, ^17^ However, this technique does not suit for the plunging ranula.^18^, ^19^, ^20^ The literature rather suggests the avoidance of the excision of the ranula itself.^2^, ^21^, ^22^ This recommendation is supported by very low recurrence rates and the most promising longterm results for the complete extirpation of the gland found in the literature.^14^, ^23^, ^24^, ^25^ However, an open surgical intervention is still related to potential complication, such as the damage of surrounding tissue like the lingual nerve or the wharton's duct.^26^

In this case report, we aim to present a rare variant of expansion of the ranula and report difficulties in diagnostics and treatment of this uncommon manifestation.

## 2 CASE REPORT

An 18‐year‐old male patient with a body mass index (BMI) of 37.4 presented with a 2 week history of progressive dysphagia and pharyngeal globus sensations. He reported difficulty in breathing, especially in the supine position, and intermittent dysphonia. Oral food intake was unrestricted. The patient reported no previous diseases, was not taking medication on a regular basis, and had no known allergies.

### 2.1 Investigations

**FIGURE 1:**
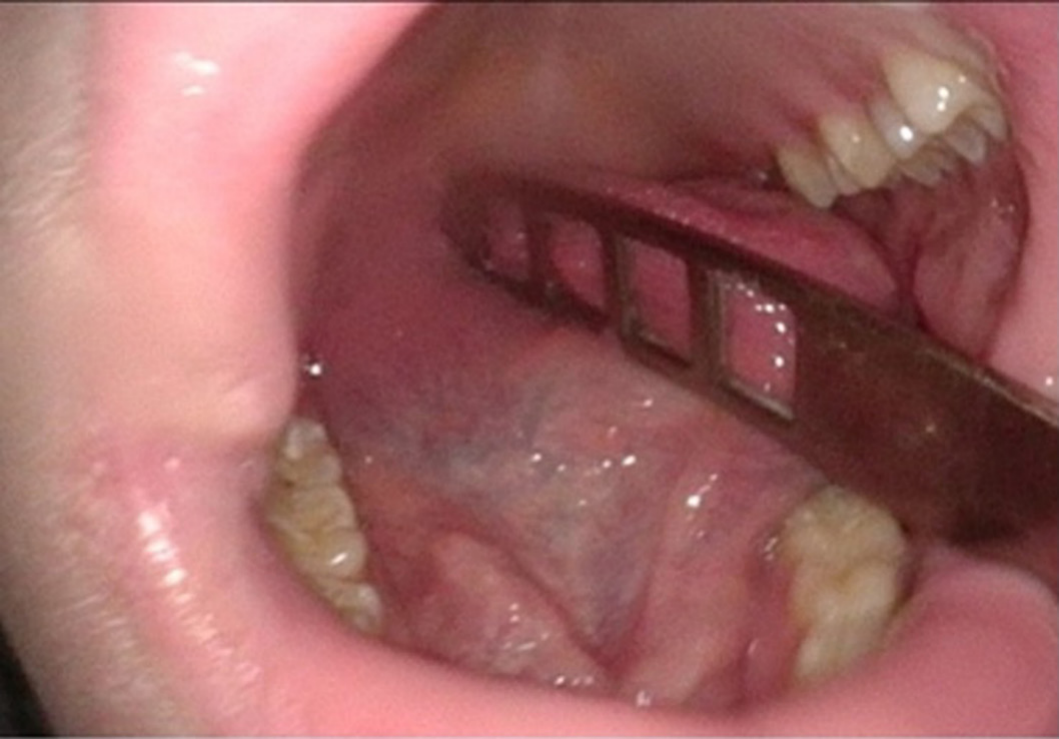
Preoperative intraoral examination showing a minimally enlarged caruncula.

**FIGURE 2:**
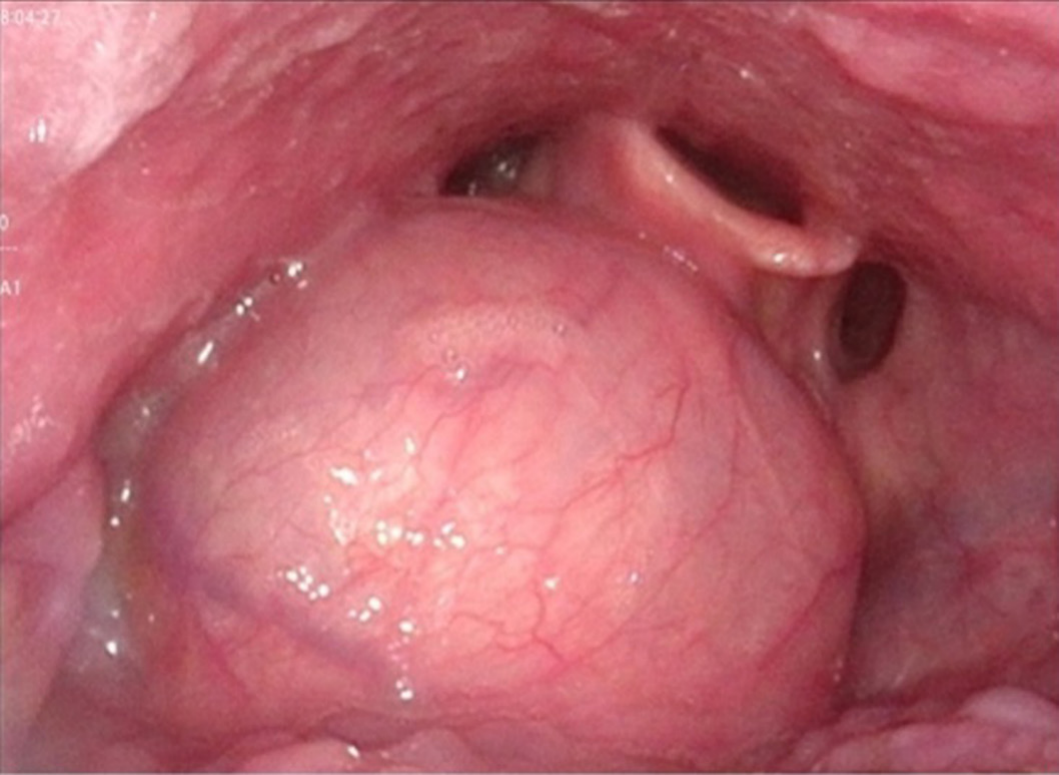
Preoperative flexible transnasal endoscopy showing a mass resembling a vallecular cyst.

ENT examination revealed a just slightly swollen floor of the oral cavity with prominent plicae (Figure 1). Flexible transnasal endoscopy revealed an extensive cystic, smooth‐edged mass in the vallecula on the right side, extending to the epiglottis and partially displacing it (Figure 2). The endolarynx could only be examined using flexible endoscopy during phonation and had regular presentation. No masses or lymphadenopathy were palpable in the neck. An expansive vallecular cyst was suspected based on the clinical presentation. As the patient had progressive symptoms and an elevated white blood cell count, direct ward admission was indicated with panendoscopy and removal of the presumably infected cyst the following day. The leucocytes were elevated to 12.4 Bn/L and the C‐reactive protein (CRP) level was 8 mg/L. No fever was present.

Intraoperatively, the cystic sac was located above the right hypopharynx and vallecular. These structures were concealed by it and thus could not be adequately assessed. In order to get a better picture of the expression, the decision to perform intraoperative ultrasound diagnostic was made. Following ultrasonography in the theater brought up the suspected diagnoses of a plunging ranula, a herniated median thyroglossal duct cyst or external laryngocele to level IB. The planned excision was not performed and the procedure was terminated for further investigations.

**FIGURE 3:**
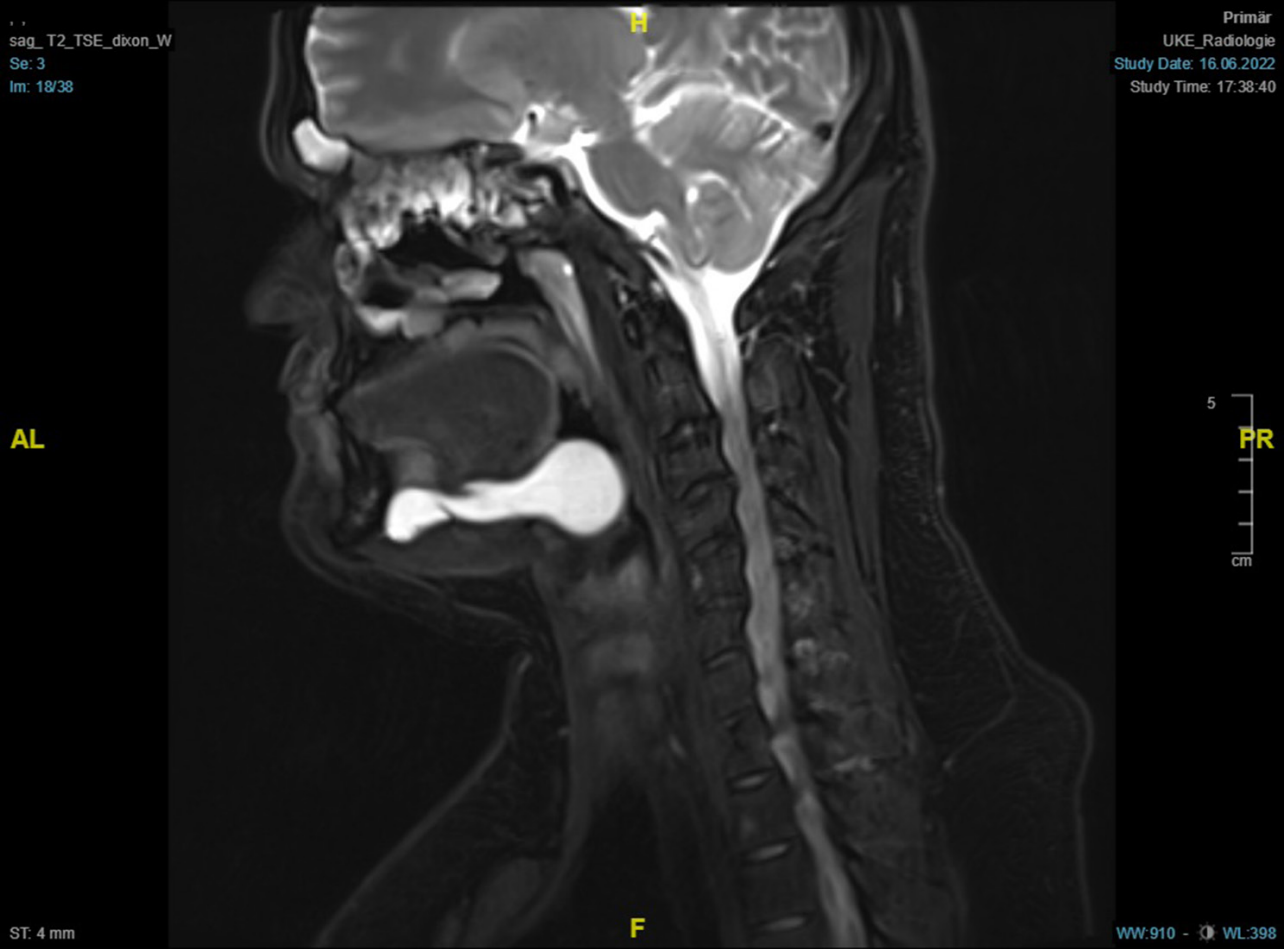
T2‐weighted contrast‐enhanced MRI of the neck (right paramedian sagittal section) showing a contrast‐absorbing mass extending posteriorly cranial to the mylohyoid muscle, reaching beyond the base of the tongue into the vallecula.

**FIGURE 4:**
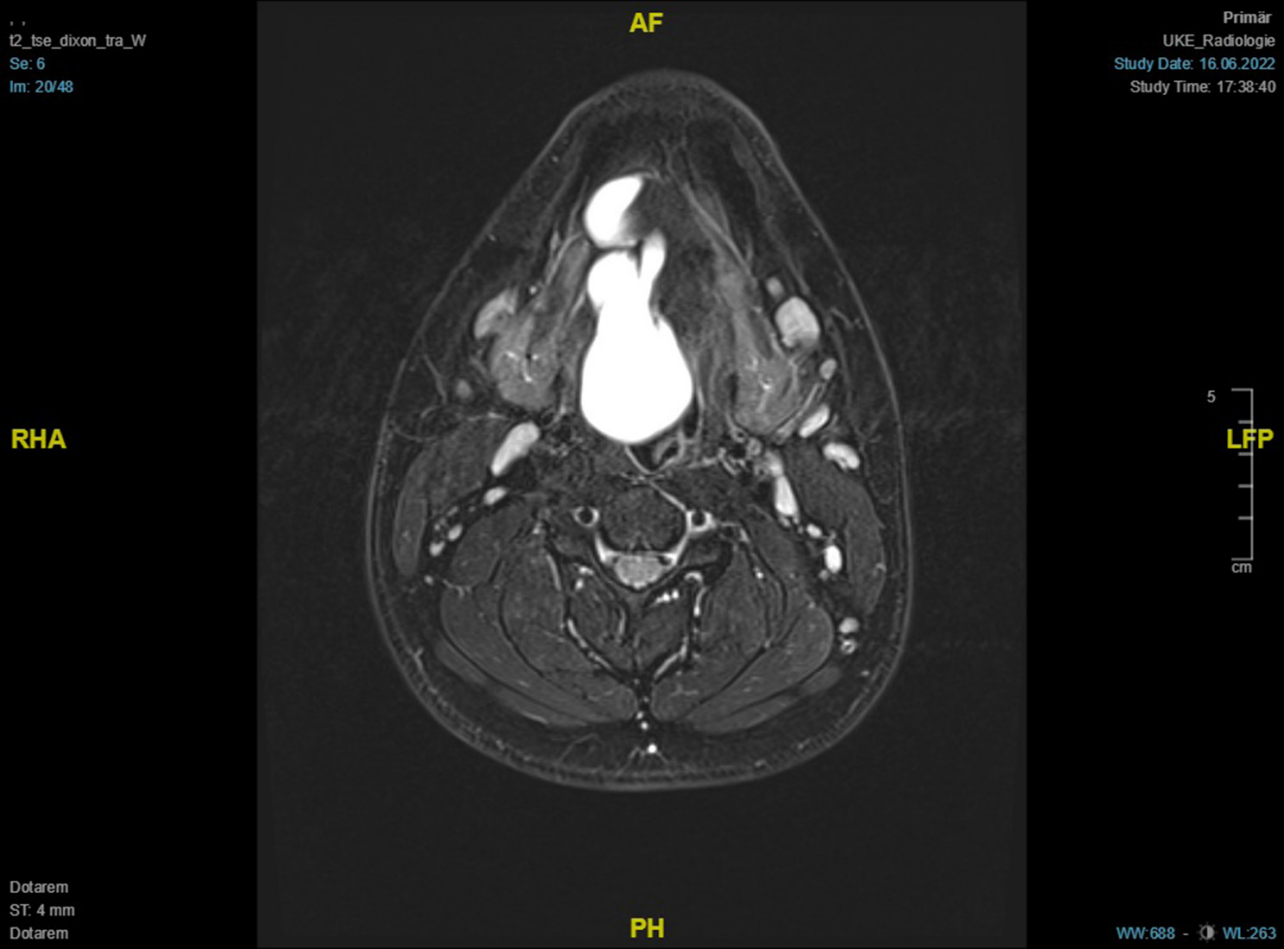
T2‐weighted contrast‐enhanced MRI of the neck (transverse section at the level of the contrast agent) showing a plunging ranula almost completely filling the floor of the oral cavity.

Following magnetic resonance imaging^27^ of the neck with contrast enhancement showed a large ranula of the right sublingual gland above the mylohyoid muscle, without contact to the submandibular gland, sliding into the ipsilateral vallecula, and with a maximum extent of 7.6 × 2.8 cm (Figures 3 and 4).

### 2.2 Treatment

**FIGURE 5:**
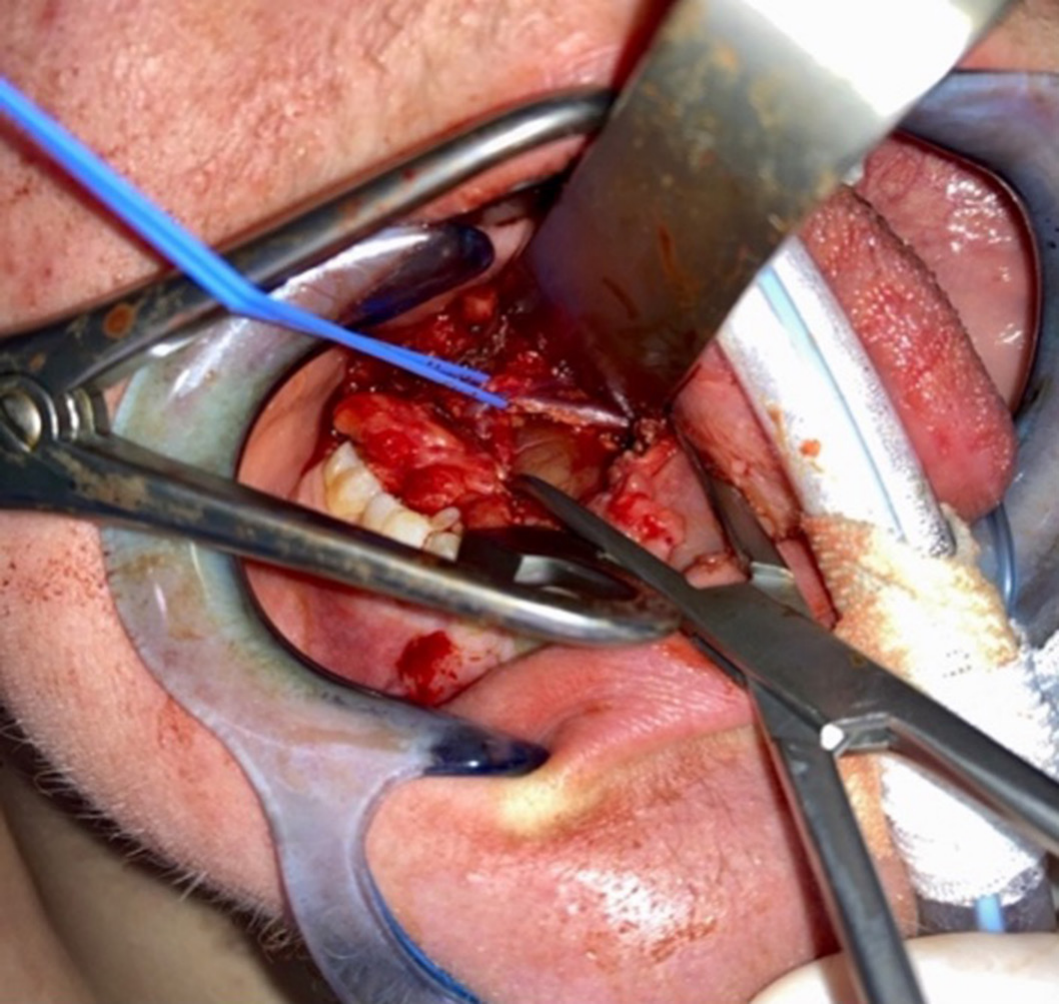
Intraoperative identification of the right sublingual nerve (entwined with a vessel loop).

**FIGURE 6:**
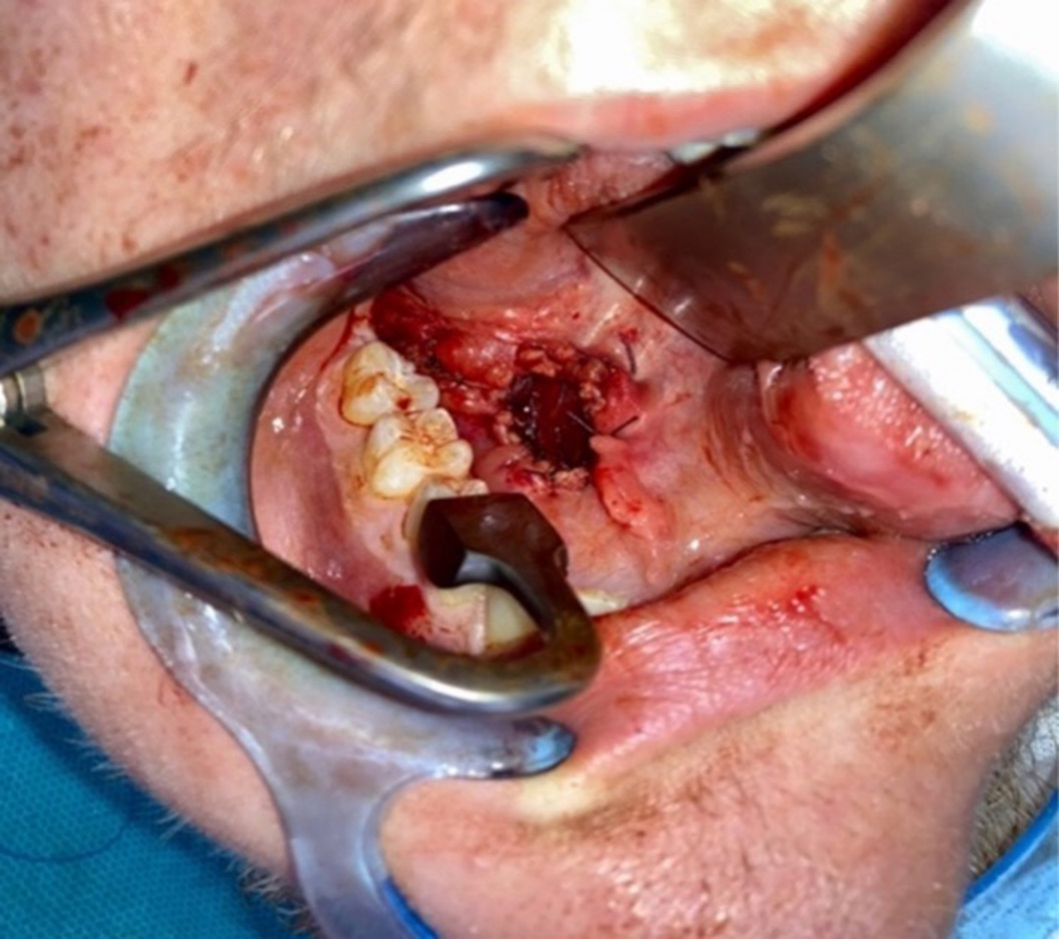
Transoral view. The final result after marsupialization of the plunging ranula, creating a large ostium allowing sufficient drainage.

Due to its extension throughout the whole base of the tongue and the patients wish for a minimally invasive procedure, we decided to perform a marsipialization. This was carried out under general anesthesia. Using a C‐shaped cheek retractor and a Denhart mouth gag, Wharton's duct on the right side was initially dilated. Probing the duct with a myrtle leaf probe was only possible up to approximately 1 cm because of stenosis. After mucosal incision, the cystic sac was carefully dissected bluntly. The lingual nerve was identified, dissected, and entwined with a vessel loop (Figure 5). The anterior part of the cyst was mobilized from the surrounding tissue, resulting in accidental opening and discharge of viscous secretions without pus. Marsupialization of the cyst sac and suturing of the wound edges with 4–0 Vicryl stitches were performed, creating a large ostium of about 1 cm (Figure 6). Examination of the base of the tongue at the end of surgery showed complete regression of the mass.

**FIGURE 7:**
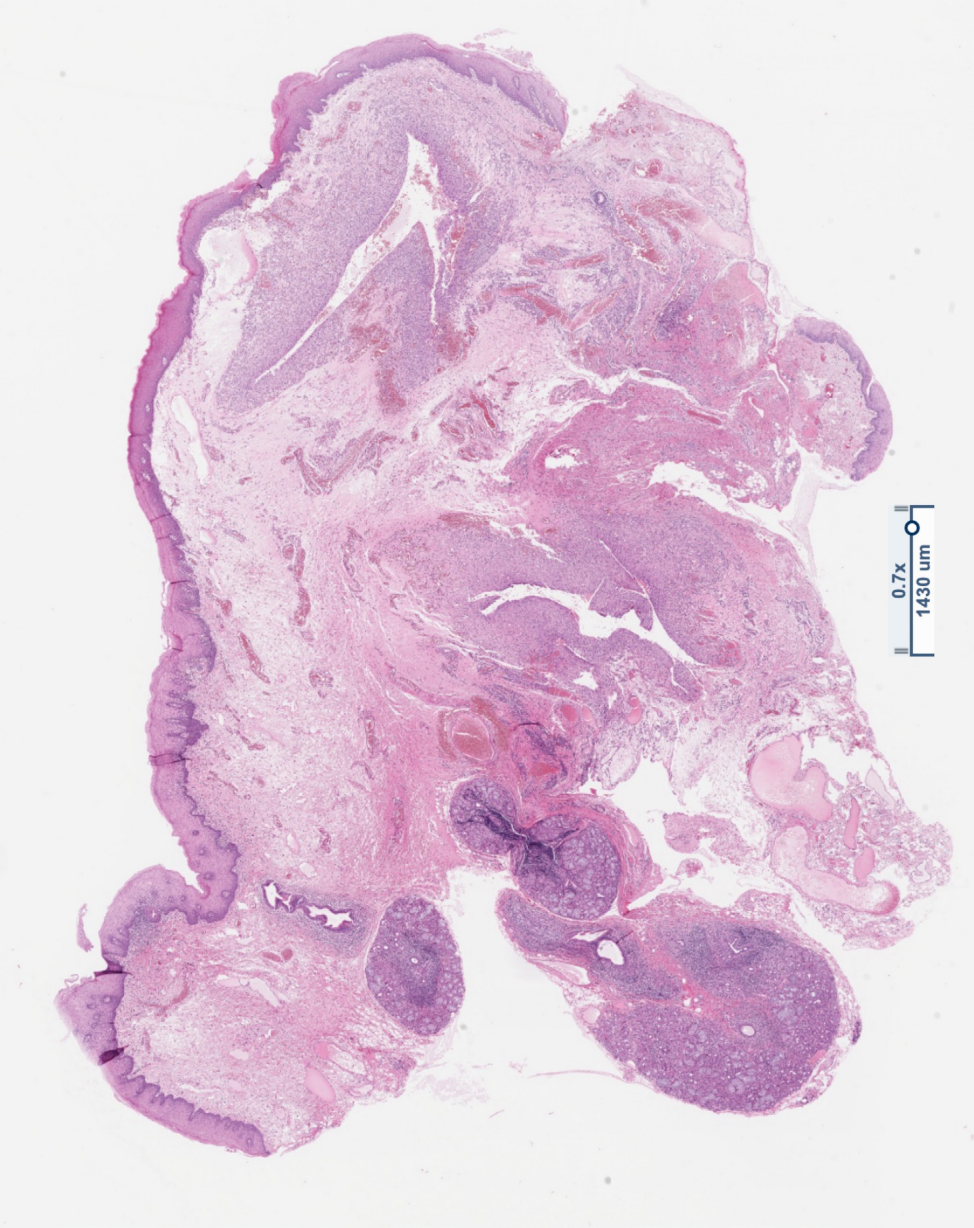
Overview image of the extirpated tissue with hematoxylin–eosin (HE) staining showing normal squamous epithelium on the surface (SE; top), sparse residual salivary gland tissue (SGT; lower left), and ranula (R; cystic structures in the center of the specimen).

**FIGURE 8:**
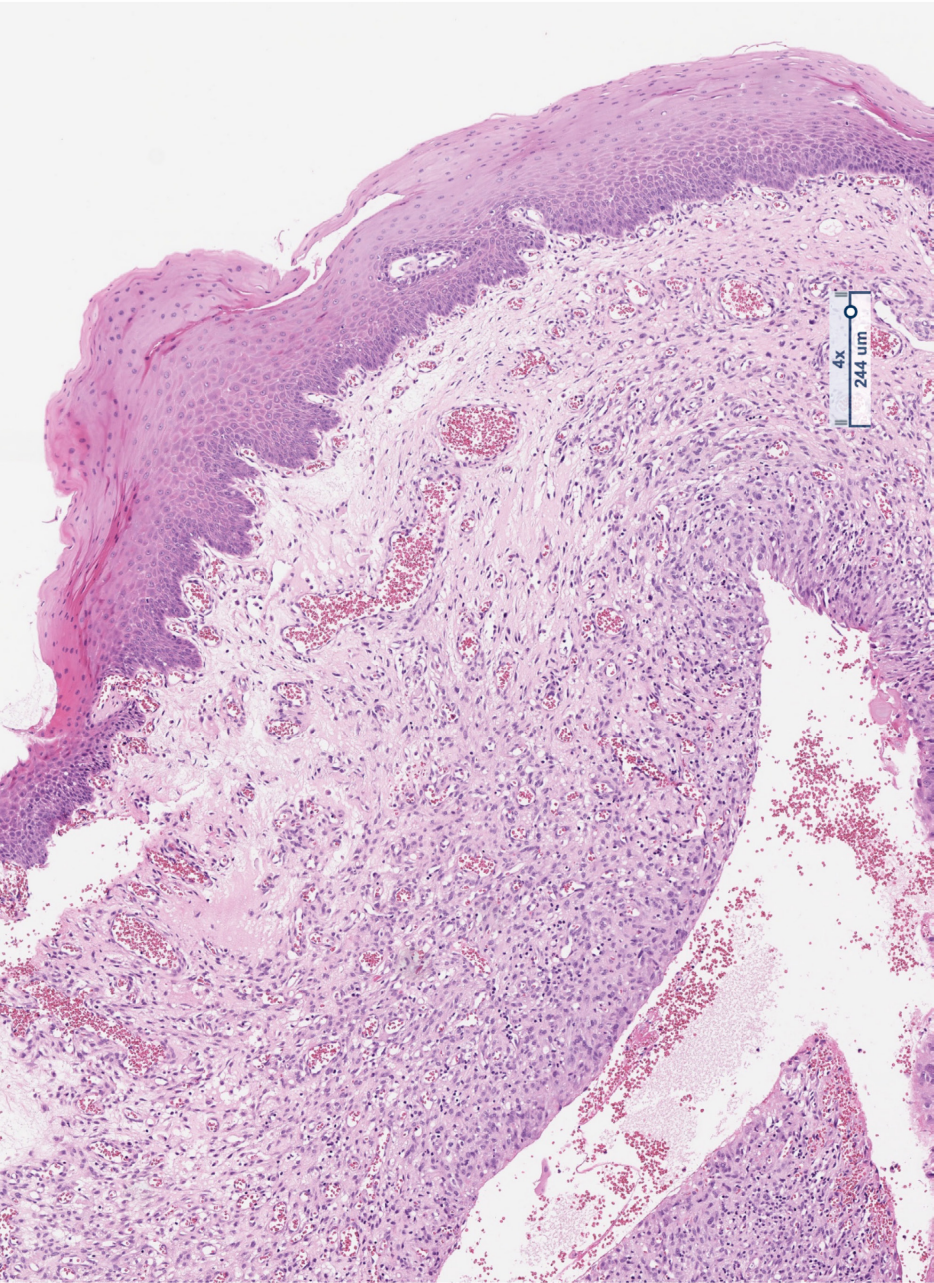
Enlarged section of the extravasation mucocele with HE‐staining. The wall shows chronic granulating inflammation. There is no epithelial lining. In the lower left corner of the image, remnants of the local squamous epithelium (SE) are still visible for orientation.

The histologically processed tissue showed findings that were clearly indicative of an extravasation mucocele of the sublingual gland with a nonepithelial lining of the cavity (Figures 7 and 8). There was mild active inflammation of the adjacent parenchyma and no evidence of malignancy.

In an outpatient follow‐up 6 month after surgery a shrinkage of the ostium and low progression of the vallecular protrusion was observed. Considering the patients will, we once more decided to surgically enlarge the ostium. Outpatient follow‐up after 8 months revealed no evidence of recurrence of the ranula itself but a still persisting ostium.

## 3 DISCUSSION

The morphology of the ranula described in our case with posterior extension of the cyst above the mylohyoid muscle posteriorly into the vallecula has not been previously reported. There only have been isolated reports of parapharyngeal extension of the formation from the supraclavicular region to the base of skull.^10^, ^28^, ^29^, ^30^, ^31^ However, since a plunging ranula is commonly defined as a mucocele passing through the mylohyoid muscle and causing a cervical swelling, we suggest the usage of the term plunging ranula with extension to the vallecula. It is an unusual clinical presentation with differing differential diagnoses to be considered than for common plunging ranulas.

The reason for this unusual expression can only be conjectured. First, there could be an embryonic disposition or stenosis^32^ supporting a slow development of the ranula over years and an exacerbation in size due to an infection. This would further explain the rapid progression of symptoms and the increased amount of white blood cells. Alternatively, an unremembered trauma of the sublingual gland could be causative for the extravasation.^3^, ^33^ As the mylohyoid muscle can be stated to be intact and without predilection site for herniation,^10^, ^31^ a deviation to the oropharyngeal region seemed to be the weakest point for extension.

As this manifestation of a ranula is new, the initial suspected diagnosis made by transnasal flexible endoscopy led to an incorrect assumption of a vallecular cyst. Following ultrasound diagnosis also failed to show a holistic picture of the expression but pointed out a large cystic formation at the floor of the mouth, which led to the decision for further investigations. For this reason, ultrasound diagnostic seems to be an effective and fast tool for the diagnosis of a plunging ranula with a cervical swelling and can pave the way for further treatment decisions.^34^ In our case, only MRI imaging was able to fully demonstrate the expansion of the cyst and was necessary for final diagnosis and treatment decision.

While preoperative imaging with ultrasound or MRI appears reasonable in the presence of a plunging ranula with a cervical component to differentiate it from other suspected diagnoses such as a lymph node enlargement, an epidermoid cyst, a lateral neck cyst, a herniated thyroglossal cyst, a cystic hygroma, or an external laryngocele,^3^, ^11^, ^16^, ^33^, ^35^ the absence of a visible cervical swelling makes differential diagnoses more difficult. However, if a massively extended vallecular cyst is present, further imaging may be useful. Therefore, it is important to differentiate patients who need further investigations from those who do not, as most patients with a suggested vallecular cyst will not have a rare manifestation of a plunging ranula. Criteria for further investigations prior to intervention may be a rapid progression of symptoms, young age, and a flexible‐endoscopically visualizable cyst that already obscures neighboring structures. Ultrasound diagnostics, fluid aspiration for amylase detection^11^, ^36^ and MRI imaging may then be critically evaluated before intervention.

Extirpation of the sublingual gland without the ranula is stated to be treatment of choice,^2^, ^26^, ^37^, ^38^ but the present patient urgently expressed the wish for the most minimally invasive therapy. We therefore decided to marsupialize the ranula. Nevertheless, this case report highlights that extensive marsupialization alone may result in a persistent ostium, only allowing longterm drainage without leading to sufficient fibrosis and healing. This does not only cause complaints but might potentially lead to recurrence in the long term. For this reason, marsupialization alone is not a sufficient therapeutic option and the result of our outpatient follow‐up indicates that the removal of the associated sublingual gland is necessary for cure.^21^

## 4 CONCLUSION

Diagnosis and therapy of this unusual manifestation of a plunging ranula with extension to the vallecula, initially presenting as a symptomatic vallecular cyst, is difficult and challenging. Even though in most cases ultrasound diagnostic is perfectly adequate, in this case, MRI imaging, was instrumental to lead to the right diagnosis. The report underlines the importance of preoperative considerations of rare diagnoses and consequences for treatment.

## AUTHOR CONTRIBUTIONS

**Lisa Schmitz:** Conceptualization; data curation; formal analysis; investigation; methodology; resources; visualization; writing – original draft. **Christian Stefan Betz:** Project administration; resources; supervision; writing – review and editing. **Franziska Büscheck:** Data curation; visualization; writing – review and editing. **Arne Böttcher:** Conceptualization; data curation; methodology; project administration; supervision; validation; writing – original draft; writing – review and editing.

## FUNDING INFORMATION

There was no financial support.

## CONFLICT OF INTEREST STATEMENT

There are no conflicts of interest to declare.

### CONSENT

Written informed consent was obtained from the patient to publish this report in accordance with the journal's patient consent policy.

## Data Availability

The data that support the findings of this study are available from the corresponding author upon reasonable request.

## References

[1] Nguyen MT , Orloff LA . Successful ablation of plunging ranula by ultrasound‐guided percutaneous ethanol injection. Laryngoscope. 2017;127(10):2239‐2241. doi:10.1002/lary.26505 28407263

[2] Harrison JD . Modern management and pathophysiology of ranula: literature review. Head Neck. 2010;32(10):1310‐1320. doi:10.1002/hed.21326 20054853

[3] Olojede ACO , Ogundana OM , Emeka CI , et al. Plunging ranula: surgical management of case series and the literature review. Clin Case Rep. 2018;6(1):109‐114. doi:10.1002/ccr3.1272 29375848 PMC5771944

[4] Aluko‐Olokun B , Olaitan AA . Ranula decompression using stitch and stab method: the Aluko technique. J Maxillofac Oral Surg. 2017;16(2):192‐196. doi:10.1007/s12663-016-0971-x 28439160 PMC5385694

[5] Langlois NE , Kolhe P . Plunging ranula: a case report and a literature review. Hum Pathol. 1992;23(11):1306‐1308. doi:10.1016/0046-8177(92)90300-r 1427758

[6] Kokong D , Iduh A , Chukwu I , Mugu J , Nuhu S , Augustine S . Ranula: current concept of pathophysiologic basis and surgical management options. World J Surg. 2017;41(6):1476‐1481. doi:10.1007/s00268-017-3901-2 28194490 PMC5422487

[7] Manna S , Bageac DV , Berenstein A , Sinclair CF , Kirke D , De Leacy R . Bleomycin sclerotherapy following doxycycline lavage in the treatment of ranulas: a retrospective analysis and review of the literature. Neuroradiol J. 2021;34(5):449‐455. doi:10.1177/19714009211008790 33832375 PMC8559021

[8] Suresh BV , Vora SK . Huge plunging ranula. J Maxillofac Oral Surg. 2012;11(4):487‐490. doi:10.1007/s12663-010-0154-0 24293946 PMC3485476

[9] de Visscher JG , van der Wal KG , de Vogel PL . The plunging ranula. Pathogenesis, diagnosis and management. J Craniomaxillofac Surg. 1989;17(4):182‐185. doi:10.1016/s1010-5182(89)80020-4 2659625

[10] AlHayek AR , Almulhem MA , Alhashim MA , Aljazan NA . Recurrent extensive plunging ranula: a rare case. J Fam Community Med. 2018;25(3):217‐219. doi:10.4103/jfcm.JFCM_24_18 PMC6130170 30220854

[11] Jain R , Morton RP , Ahmad Z . Diagnostic difficulties of plunging ranula: case series. J Laryngol Otol. 2012;126(5):506‐510. doi:10.1017/S0022215112000230 22401594

[12] Jain P . Plunging ranulas and prevalence of the “tail sign” in 126 consecutive cases. J Ultrasound Med. 2020;39(2):273‐278. doi:10.1002/jum.15100 31334858

[13] Morton RP , Ahmad Z , Jain P . Plunging ranula: congenital or acquired? Otolaryngol Head Neck Surg. 2010;142(1):104‐107. doi:10.1016/j.otohns.2009.10.014 20096232

[14] Elnager M , Udeabor SE , Elfadeel ASA , Onwuka CI , Hamid MMM , Alsubaie YMA . Modified micromarsupialization technique as an alternative primary treatment for ranulas: a case series in a resource‐challenged economy. Clin Exp Dent Res. 2022;8:1434‐1439. doi:10.1002/cre2.627 36196590 PMC9760142

[15] Choi MG . Case report of the management of the ranula. J Korean Assoc Oral Maxillofac Surg. 2019;45(6):357‐363. doi:10.5125/jkaoms.2019.45.6.357 31966981 PMC6955425

[16] Lyly A , Castren E , Aronniemi J , Klockars T . Plunging ranula— patient characteristics, treatment, and comparison between different populations. Acta Otolaryngol. 2017;137(12):1271‐1274. doi:10.1080/00016489.2017.1357082 28754079

[17] Mortellaro C , Dall'Oca S , Lucchina AG , et al. Sublingual ranula: a closer look to its surgical management. J Craniofac Surg. 2008;19(1):286‐290. doi:10.1097/SCS.0b013e31815ca1cd 18216704

[18] Yoshimura Y , Obara S , Kondoh T , Naitoh S . A comparison of three methods used for treatment of ranula. J Oral Maxillofac Surg. 1995;53(3):280‐282. doi:10.1016/0278-2391(95)90224-4 7861278

[19] Haberal I , Gocmen H , Samim E . Surgical management of pediatric ranula. Int J Pediatr Otorhinolaryngol. 2004;68(2):161‐163. doi:10.1016/j.ijporl.2003.09.017 14725982

[20] Mizuno A , Yamaguchi K . The plunging ranula. Int J Oral Maxillofac Surg. 1993;22(2):113‐115. doi:10.1016/s0901-5027(05)80815-x 8320447

[21] Morton RP . Surgical management of ranula revisited. World J Surg. 2018;42(9):3062‐3063. doi:10.1007/s00268-018-4666-y 29750326

[22] Harrison JD . The persistently misunderstood plunging ranula. Am J Otolaryngol. 2022;43(1):103276. doi:10.1016/j.amjoto.2021.103276 34763952

[23] Patel MR , Deal AM , Shockley WW . Oral and plunging ranulas: what is the most effective treatment? Laryngoscope. 2009;119(8):1501‐1509. doi:10.1002/lary.20291 19504549 PMC4455536

[24] Lomas J , Chandran D , Whitfield BCS . Surgical management of plunging ranulas: a 10‐year case series in South East Queensland. ANZ J Surg. 2018;88(10):1043‐1046. doi:10.1111/ans.14356 29266658

[25] Chung YS , Cho Y , Kim BH . Comparison of outcomes of treatment for ranula: a proportion meta‐analysis. Br J Oral Maxillofac Surg. 2019;57(7):620‐626. doi:10.1016/j.bjoms.2019.06.005 31239229

[26] Zhao YF , Jia J , Jia Y . Complications associated with surgical management of ranulas. J Oral Maxillofac Surg. 2005;63(1):51‐54. doi:10.1016/j.joms.2004.02.018 15635557

[27] Abouhanine O , Merzem A , Ndayishimiye V , et al. Sinonasal chondrosarcoma, an unusual location. Eur J Case Rep Intern Med. 2020;7(12):001933. doi:10.12890/2020_001933 33457352 PMC7806288

[28] Clyburn VL 3rd , Smith JE , Rumboldt T , Matheus MG , Day TA . Ascending and plunging ranula in a pediatric patient. Otolaryngol Head Neck Surg. 2009;140(6):948‐949. doi:10.1016/j.otohns.2008.12.041 19467424

[29] Shelley MJ , Yeung KH , Bowley NB , Sneddon KJ . A rare case of an extensive plunging ranula: discussion of imaging, diagnosis, and management. Oral Surg Oral Med Oral Pathol Oral Radiol Endod. 2002;93(6):743‐746. doi:10.1067/moe.2002.122504 12142883

[30] Kumbul YC , Okur N , Ciris IM , Okur E , Sivrice ME , Akin V . A giant diving ranula extending to the skull base in pediatric age. J Craniofac Surg. 2021;32(5):e515‐e517. doi:10.1097/SCS.0000000000007527 34319685

[31] Zhi K , Gao L , Ren W . What is new in management of pediatric ranula? Curr Opin Otolaryngol Head Neck Surg. 2014;22(6):525‐529. doi:10.1097/MOO.0000000000000103 25211709

[32] Borkar NB , Mohanty D , Hussain N , Dubey R , Singh S , Varshney A . A rare case of congenital ranula. Afr J Paediatr Surg. 2021;18(2):106‐108. doi:10.4103/ajps.AJPS_36_20 33642410 PMC8232361

[33] Kalra V , Mirza K , Malhotra A . Plunging ranula. J Radiol Case Rep. 2011;5(6):18‐24. doi:10.3941/jrcr.v5i6.682 PMC3303342 22470797

[34] Jain P , Jain R , Morton RP , Ahmad Z . Plunging ranulas: high‐resolution ultrasound for diagnosis and surgical management. Eur Radiol. 2010;20(6):1442‐1449. doi:10.1007/s00330-009-1666-1 19943050

[35] Gupta A , Karjodkar FR . Plunging ranula: a case report. ISRN Dent. 2011;2011:806928. doi:10.5402/2011/806928 21991487 PMC3169347

[36] Song T , Chiu W , de Paiva LS , et al. Amylase as a diagnostic tool for plunging ranula: clinical series and description of the technique. Laryngoscope. 2023;133(3):535‐538. doi:10.1002/lary.30243 35670504

[37] Zhao YF , Jia Y , Chen XM , Zhang WF . Clinical review of 580 ranulas. Oral Surg Oral Med Oral Pathol Oral Radiol Endod. 2004;98(3):281‐287. doi:10.1016/S1079210404000800 15356464

[38] Syebele K , Munzhelele TI . The anatomical basis and rational for the transoral approach during the surgical excision of the sublingual salivary gland for the management of plunging ranula. Am J Otolaryngol. 2020;41(2):102371. doi:10.1016/j.amjoto.2019.102371 31917022

